# Effect of intravenous immunoglobulin on mortality in hospitalized patients with COVID‐19: A systematic review and meta‐analysis of randomized controlled trials

**DOI:** 10.1002/hsr2.2239

**Published:** 2024-07-08

**Authors:** Dinesh Sangarran Ramachandram, Chia Siang Kow, Syed Shahzad Hasan, Kaeshaelya Thiruchelvam

**Affiliations:** ^1^ School of Pharmacy Monash University Malaysia Bandar Sunway Selangor Malaysia; ^2^ School of Pharmacy IMU University Kuala Lumpur Malaysia; ^3^ School of Applied Sciences University of Huddersfield Huddersfield United Kingdom

**Keywords:** COVID‐19, immunoglobulin, IVIG, mortality, safety

## Abstract

**Background and Aims:**

We performed a meta‐analysis of randomized controlled trials (RCTs) to summarize the overall effect of intravenous immunoglobulin (IVIG) on mortality outcomes among hospitalized coronavirus disease 2019 (COVID‐19) patients.

**Methods:**

We systematically searched electronic databases up to June 1, 2023. Pooled odds ratio (OR) of mortality with a 95% confidence interval (CI) was generated using a random‐effects model. The risk of bias was appraised using the Cochrane risk‐of‐bias Version 2 tool for randomized trials.

**Results:**

Nine RCTs were included: three RCTs had an overall low risk of bias, four RCTs had some concerns in the overall risk of bias, and two RCTs trials had an overall high risk of bias. The use of IVIG indicated a significant reduction in the odds of mortality (pooled OR = 0.69; 95% CI 0.50–0.96) relative to nonuse of IVIG. Subgroup analysis in patients with a severe course of COVID‐19 revealed no significant reduction in the odds of mortality (pooled OR = 0.58; 95% CI 0.29–1.16).

**Conclusions:**

We suggest exercising caution when interpreting effectiveness of IVIG in reducing mortality among hospitalized patients with COVID‐19. Our findings emphasize for larger trials with rigorous study designs to better understand the impact of IVIG, particularly in those with severe COVID‐19.

## Key points

- The use of intravenous immunoglobulin (IVIG) indicated a significant reduction in the odds of mortality relative to nonuse of IVIG.
- Subgroup analysis in patients with a severe course of coronavirus disease 2019 (COVID‐19) revealed no significant reduction in the odds of mortality.
- We suggest exercising caution when interpreting the efficacy of IVIG in reducing mortality among hospitalized patients with COVID‐19.

## 1 INTRODUCTION

Intravenous immune globulin (IVIG) is a blood product derived from the pooled serum or plasma of thousands of healthy paid donors. The primary component of IVIG is the serum IgG fraction, mainly consisting of the IgG1 and IgG2 subclasses.^1^ Therefore, the administration of IVIG in the context of immunodeficiency states could provide passive immunity with adequate concentrations of antibodies against a broad range of pathogens. Nevertheless, the decision to repurpose IVIG for the treatment of coronavirus disease 2019 (COVID‐19) is based on its several potential anti‐inflammatory and immunomodulatory effects,^2^, ^3^, ^4^ since we have currently acknowledged that hyperactive immune response can occur in a certain fraction of patients with COVID‐19.^5^

Positive outcomes have been observed using IVIG in clinical studies involving COVID‐19 patients. A recent systematic review and meta‐analysis by Xiang et al.^6^ examined the efficacy of IVIG in treating COVID‐19 patients. This meta‐analysis, which included seven clinical studies (three observational), found that IVIG administration was associated with significant mortality benefits compared to control treatment (pooled risk ratio = 0.67; 95% confidence interval [CI] 0.52−0.86).

Nevertheless, a meta‐analysis of observational studies, especially with a retrospective design, could introduce bias since the efficacy estimates may not be adjusted with important confounders. Therefore, the reported mortality benefits of IVIG in COVID‐19 patients should be interpreted with caution. Several randomized controlled trials (RCTs) have assessed the impact of IVIG on mortality outcomes in COVID‐19 patients. Our objective was to conduct a systematic review and meta‐analysis to summarize its effect based on randomized evidence.

## 2 METHODS

This study followed the guidelines outlined in the Preferred Reporting Items for Systematic Reviews and Meta‐Analyses (PRISMA).^7^ We conducted a systematic search of electronic databases, including PubMed, Scopus, and the Cochrane Central Register of Controlled Trials, as well as preprint repositories (medRxiv and SSRN), from their inception until June 1, 2023. The search included the following keywords and their MeSH terms: “COVID‐19,” “SARS‐CoV‐2,” “novel coronavirus disease,” “intravenous immunoglobulin,” “intravenous immune globulin,” “IVIG,” “immunotherapy,” “randomized,” “controlled trial,” and “clinical trial.” Additionally, we searched the United States Clinical Trial Registries (clinicaltrials.gov) for ongoing registered clinical trials of IVIG in the treatment of COVID‐19 that had released findings. Additionally, we manually examined the reference lists of relevant articles to identify further studies.

Our inclusion criteria focused on RCTs that compared the mortality outcomes of IVIG with those of comparators in hospitalized COVID‐19 patients. We excluded single‐arm trials, non‐randomized trials, and trials that did not report mortality events. The primary outcome of interest was all‐cause mortality.

Two authors (C. S. K. and D. S. R.) independently assessed each included trial, extracting key study characteristics using a pre‐designed data extraction form. The extracted data encompassed the first author's surname, the design of the trial, the country where the study was conducted, the age of the participants, the proportion of patients requiring respiratory support at baseline, the IVIG regimen, comparative agents, and mortality events. The risk of bias in the included trials was assessed by two authors (C. S. K. and S. S. H.) using Version 2 of the Cochrane risk‐of‐bias tool for randomized trials (RoB 2).^8^ This tool evaluates potential bias across several domains, including randomization, deviations from intervention, missing outcome data, measurement of outcomes, and selection of reported results. Each domain was judged as “Low,” “High,” or “Some concerns” for risk of bias. An overall low risk of bias indicated high methodological quality, some concerns indicated potential issues in one or more domains, and a high risk of bias indicated significant methodological deficiencies that might affect the reliability of the results.

For the meta‐analysis, we used a random‐effects model to pool mortality estimates from individual trials, presenting the results as pooled odds ratios (OR) with 95% confidence intervals (CIs). We assessed heterogeneity using *I*² statistics and the *χ*² test, with thresholds for statistical significance set at 50% and *p* < 0.10, respectively. To ensure the robustness of our findings, we performed a sensitivity analysis by systematically excluding trials with a high risk of bias. This analysis aimed to determine if the main results were consistent when higher‐risk studies were removed. Additionally, we conducted a subgroup analysis to explore the effect of IVIG on mortality within specific subpopulations, particularly focusing on trials that included only hospitalized patients with severe COVID‐19. This subgroup analysis aimed to identify whether the mortality benefits of IVIG were more pronounced in this subset of patients. All analyses were conducted using Meta XL, version 5.3 (EpiGear International).

## 3 RESULTS

**Figure 1:**
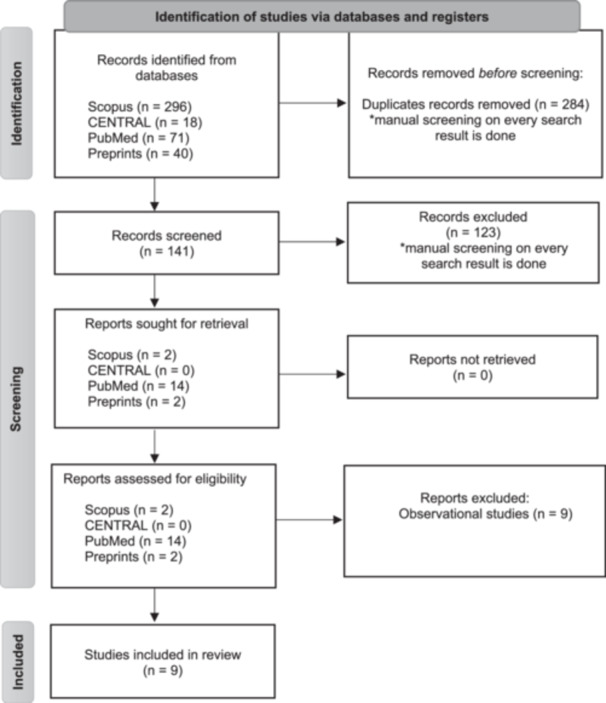
Flow diagram of study selection.

Our comprehensive literature search yielded 425 records, with 141 being unique entries. After a thorough screening process, we included nine RCTs^9^, ^10^, ^11^, ^12^, ^13^, ^14^, ^15^, ^16^, ^17^ in the analysis. These trials involved a total of 748 patients receiving IVIG and 682 patients in the control group. The study selection process and review flowchart are illustrated in Figure 1.

The included trials were conducted in various countries: Iran (*n* = 2),^9^, ^12^ India (*n* = 2),^10^, ^14^ the United States (*n* = 1),^11^ Pakistan (*n* = 1),^13^ France (*n* = 1),^15^ Israel (*n* = 1),^16^ with one multinational trial, INSIGHT 013, spanning 63 sites in 11 countries.^17^ Table 1 provides details of these studies.

**Table 1:** Study characteristics of included trials.

**Study**	**Study design**	**Country**	**Age (median/mean)**	**Definition of severe illness**	**Mean/median C‐reactive protein level at baseline (mg/L)**	**Regimen of IVIG in the intervention group**	**Regimen of comparator intervention in the control group**	**Mortality events**	
								**IVIG users (*n*/*N*; %)**	**Non‐IVIG users (*n*/*N*; %)**
Gharebaghi et al.^9^	Randomized, double‐blind, placebo‐controlled trial	Iran	IVIG users = 55.5 Non‐IVIG users = 56.0	Oxygen saturation <90%	Not reported	5 g four times a day for 3 consecutive days	Placebo	6/30; 20.0	14/29; 48.3
Raman et al.^10^	Open‐label, randomized controlled trial	India	IVIG users = 48.4 Non‐IVIG users = 49.0	N/A (enrolled patients had non‐severe illness)	IVIG users = 24.1 Non‐IVIG users = 29.8	0.4 g/kg once a day for 5 consecutive days (plus standard of care)	Standard of care: azithromycin, lopinavir/ritonavir, piperacillin + tazobactam, acetaminophen, pantoprazole	0/50; 0	1/49; 2.0
Sakoulas et al.^11^	Open‐label, randomized controlled trial	United States	IVIG users = 54.0 Non‐IVIG users = 54.0	Oxygen saturation ≤96% while on ≥4 L/min oxygen therapy by nasal cannula	IVIG users = 142.0 Non‐IVIG users = 140.0	0.5 g/kg once a day for 3 consecutive days (plus standard of care)	Standard of care: remdesivir, convalescent plasma, and/or glucocorticoids	1/16; 6.3	3/17; 17.6
Tabarsi et al.^12^	Open‐label, randomized controlled trial	Iran	IVIG users = 54.3 Non‐IVIG users = 52.5	Respiratory rates ≥30 breaths/min, oxygen saturation ≤93%, and/or ratio of arterial oxygen partial pressure to fractional inspired oxygen ≤300 mmHg	IVIG users (survived) = 39.0 IVIG users (not survived) = 62.0 Non‐IVIG users (survived) = 52.5 Non‐IVIG users (survived) = 56.5	0.4 g/kg once a day for 3 consecutive days (plus standard of care)	Standard of care: Oral lopinavir/ritonavir 400/100 mg twice a day and hydroxychloroquine 200 mg twice a day	24/52; 46.2	14/32; 43.8
Ali et al.^13^	Randomized, slingle‐blind, placebo‐controlled trial	Pakistan	IVIG users = 55.9 Non‐IVIG users = 59.1	Requirement for supplemental oxygen, noninvasive ventilation, high‐flow oxygen therapy, or invasive ventilation	IVIG users = 99.9 Non‐IVIG users = 104.1	0.15, 0.2, 0.25, or 0.3 g/kg as a single dose	Intravenous remdesivir 200 mg as loading dose, then 100 mg once daily for 5 days, enoxaparin, and glucocorticoids (oral dexamethasone 6 mg once daily or intravenous methylprednisolone 0.5−1 mg/kg twice daily)	10/40; 25.0	6/10; 60.0
Parikh et al.^14^	Open‐label, randomized controlled trial	India	IVIG users = 53.0 Non‐IVIG users = 52.0	N/A (enrolled patients had non‐severe illness)	IVIG users = 83.1 Non‐IVIG users = 56.4	COVID‐19 hyperimmune IVIG (350 AU/mL) 30 mL for 2 consecutive days (plus standard of care)	Standard of care	1/30; 3.3	1/30; 3.3
Mazeraud et al.^15^	Randomized, double‐blind, placebo‐controlled trial	France	IVIG users = 65.1 Non‐IVIG users = 66.5	Moderate or severe COVID‐19‐associated acute respiratory distress syndrome requiring mechanical ventilation	IVIG users = 164.0 Non‐IVIG users = 160.0	0.5 g/kg once a day for 4 consecutive days	Placebo	28/69; 40.6	31/77; 40.3
INSIGHT 013 Study Group^17^	Randomized, double‐blind, placebo‐controlled trial	11 countries	IVIG users = 58 Non‐IVIG users = 60	N/A (not all enrolled patients had severe illness, and subgroup analysis was not performed on mortality outcomes)	IVIG users = 61.0 Non‐IVIG users = 63.0	COVID‐19 hyperimmune IVIG 400 mg/kg as a single dose	Placebo	18/295; 6.1	22/284; 7.8
Maor et al.^16^	Open‐label, randomized controlled trial	Israel	IVIG users = 64.0 Non‐IVIG users = 66.0	N/A (not all enrolled patients had severe illness, and subgroup analysis was not performed on mortality outcomes)	IVIG users = 11.6 Non‐IVIG users = 14.8	4 g daily for 2 consecutive days	Convalescent plasma	16/166;9.6	23/153;15.0

Abbreviation: IVIG, intravenous immune globulin.

The IVIG regimens varied across the nine trials.^9^, ^10^, ^11^, ^12^, ^13^, ^14^, ^15^, ^16^, ^17^ Gharebhagi et al.^9^ administered 5 g intravenously four times a day for 3 days. Sakoulas et al.^11^ used 0.5 g/kg once daily for 3 days. Tabarsi et al.^12^ and Raman et al.^10^ administered 0.4 g/kg once daily for 3 and 5 days, respectively. Ali et al.^13^ administered one of four dosages (0.15, 0.2, 0.25, and 0.3 g/kg) as a single dose. Parikh et al.^14^ used COVID‐19 hyperimmune globulin (350 AU/mL) as a 30 mL intravenous infusion for 2 days. Mazeraud et al.^15^ administered 0.5 g/kg once daily for 4 days. INSIGHT 013^17^ used COVID‐19 hyperimmune IVIG at 400 mg/kg as a single dose, and Maor et al.^16^ administered 4 g daily for 2 days.

The overall risk of bias, as assessed by RoB 2, is presented in Table 2. Mazeraud et al.,^15^ INSIGHT 013,^17^ and Maor et al.^16^ had a low risk of bias across all domains. Four trials had some concerns regarding bias: Sakoulas et al.^11^ had unclear allocation concealment and an open‐label design; Tabarsi et al.^12^ had unclear allocation concealment, an open‐label design, and lacked a trial protocol/statistical analysis plan; Raman et al.^10^ had an open‐label design and lacked a trial protocol/statistical analysis plan; Ali et al.^13^ had unblinded personnel/carers.

**Table 2:** Risk of bias of included trials.

	Gharebaghi et al.^9^	Raman et al.^10^	Sakoulas et al.^11^	Tabarsi et al.^12^	Ali et al.^13^	Parikh et al.^14^	Mazeraud et al.^15^	INSIGHT 013 Study Group^17^	Maor et al.^16^
Randomization	Some concerns	Low risk	Some concerns	Some concerns	Low risk	High risk	Low risk	Low risk	Low risk
Deviations from intervention	Low risk	Some concerns	Some concerns	Some concerns	Some concerns	Some concerns	Low risk	Low risk	Low risk
Missing outcome data	High risk	Low risk	Low risk	Low risk	Low risk	Low risk	Low risk	Low risk	Low risk
Measurement of the outcome	Low risk	Low risk	Low risk	Low risk	Low risk	Low risk	Low risk	Low risk	Low risk
Selection of the reported results	Low risk	Some concerns	Low risk	Some concerns	Low risk	Low risk	Low risk	Low risk	Low risk
Overall risk of bias	High risk	Some concerns	Some concerns	Some concerns	Some concerns	High risk	Low risk	Low risk	Low risk

Two trials had a high risk of bias: Gharebaghi et al.^9^ had missing outcome data and unclear randomization details, and Parikh et al.^14^ had baseline biomarker differences and an open‐label design.

**Figure 2:**
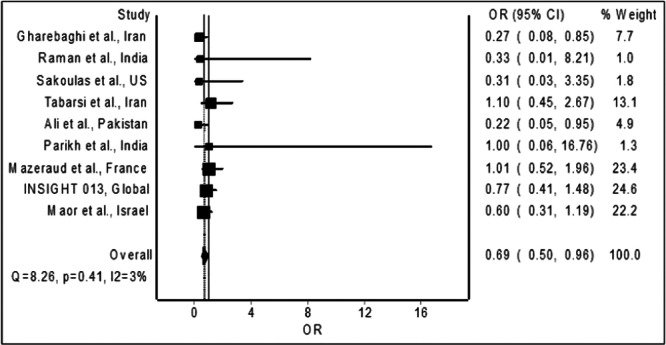
Forest plot showing the pooled odds ratio of mortality among hospitalized patients with COVID‐19 treated with IVIG compared to those not treated with IVIG. COVID‐19, coronavirus disease 2019; IVIG, intravenous immunoglobulin.

The meta‐analysis of the nine trials^9^, ^10^, ^11^, ^12^, ^13^, ^14^, ^15^, ^16^, ^17^ revealed a significant reduction in the odds of mortality among hospitalized COVID‐19 patients receiving IVIG compared to the control group (Figure 2; pooled OR = 0.69; 95% CI 0.50−0.96), providing sufficient evidence to reject the null hypothesis of no significant difference. Sensitivity analysis, excluding trials with a high risk of bias, showed no significant mortality reduction (pooled OR = 0.75; 95% CI 0.53−1.05). Similarly, a subgroup analysis of trials^9^, ^11^, ^12^, ^13^, ^15^ involving only hospitalized patients with severe COVID‐19 did not show significant mortality benefits (pooled OR = 0.58; 95% CI 0.29−1.16) with IVIG administration.

## 4 DISCUSSION

To the best of the authors' knowledge, this is the most current systematic review and meta‐analysis of RCTs investigating the effect of IVIG on mortality outcomes in COVID‐19 patients. Our meta‐analysis, which focuses on RCTs—the gold standard for assessing causal relationships between interventions and outcomes—demonstrates that IVIG administration significantly reduced the risk of mortality in hospitalized COVID‐19 patients.

Our findings concur with real‐world observations. Shao et al.,^18^ in their multicentre retrospective cohort study, which investigated the efficacy of IVIG (0.1−0.5 g/kg/day for the duration of 5−15 days) in 325 severely and critically ill hospitalized patients with COVID‐19, reported that that the administration of IVIG is associated with mortality benefits (hazard ratio = 0.40; 95% CI 0.20−0.80) in hospitalized patients critically ill with COVID‐19. In addition, in a single‐center retrospective cohort study by Esen et al.,^19^ involving 93 hospitalized patients with severe COVID‐19, it was reported that IVIG treatment was associated with a significantly prolonged median survival time (68 days compared to 18 days; *p* = 0.014). Although observational studies are notoriously associated with biases, which may question the implications of their findings, our meta‐analysis of RCTs resulted in robust conclusions which support these findings.

Our study has important implications in the management of hospitalized COVID‐19 patients globally. The use of IVIG has been associated with beneficial outcomes and we confirm the mortality reduction among hospitalized patients with COVID‐19 treated with IVIG. The mechanisms behind the clinical benefits of IVIG are not fully understood. However, IVIG has the potential to mitigate cytokine storms in COVID‐19 patients by scavenging complements, inhibiting innate immune cells and effector T‐cell activation, and expanding regulatory T cells, which is evident in the reduction in the inflammatory mediators following IVIG therapy^18^; the trial by Ali et al.^13^ showed that the decrement in C‐reactive protein level in the intervention arm (45.5−87.4 mg/L) was more than that of the control arm (18.3 mg/L) after 24 h of IVIG therapy; and the trial by Sakoulas et al.^11^ demonstrated that IVIG therapy reduced interleukin‐6 serum concentrations, with the IVIG group showing a median of 5 pg/mL compared to the control group's median of 18 pg/mL after 24−48 h of treatment. Therefore, future trials could target only hospitalized patients with COVID‐19 and biochemical evidence of inflammation to demonstrate mortality benefits.

One aspect that requires attention is the impact of IVIG on specific patient populations, particularly those with severe COVID‐19. The subgroup analysis conducted on trials^9^, ^11^, ^12^, ^13^, ^15^ which focused on hospitalized patients with severe COVID‐19, did not demonstrate significant mortality benefits associated with IVIG administration relative to nonuse of IVIG. Nevertheless, it should be noted that the definition of severe illness differs across the included trials, which makes it challenging to draw definitive conclusions regarding the efficacy of IVIG in this specific patient population. Within the existing trials, there is an individual analysis worth considering. The trial reported by Gharebaghi et al.^9^ that recruited patients with severe COVID‐19, defined as those with oxygen saturation levels below 90%, observed a significant reduction in mortality with IVIG administration. This finding suggests that IVIG might be more effective in patients with a specific severity criterion, such as low oxygen saturation levels. These results highlight the importance of conducting further randomized trials to address the gap in knowledge regarding the efficacy of IVIG in patients with severe COVID‐19. Standardizing the definition of severe illness across trials would enable more reliable comparisons and conclusions.

Adverse reactions to IVIG are a concern since they are reported to affect up to 50% of patients receiving IVIG before the COVID‐19 pandemic.^20^, ^21^, ^22^, ^23^, ^24^, ^25^, ^26^, ^27^ Previous literature observed that headache is a common side effect of IVIG. In fact, the headache was reported as an adverse event in the trial by Raman et al.^10^ though the frequency of occurrence was not available. Furthermore, IVIG infusions can be associated with thromboembolic events. Consequently, all IVIG products carry a Boxed Warning in their prescribing information to alert healthcare providers about this potential risk.^28^ This is of concern since COVID‐19 itself has been associated with hypercoagulability.^29^

The trial reported by Mazeraud et al.^15^ observed thromboembolic events among the participants where the intervention arm had a higher rate of thromboembolic events compared to the control arm (15% vs. 4%). Such findings demand attention from the clinicians who are intended to administer IVIG to their patients with COVID‐19. Nevertheless, the trial reported by Maor et al. observed a comparable rate of thromboembolic events between the intervention arm and the control arm (2% vs. 3%). On the other hand, eczematous dermatitis can be associated with IVIG therapy,^30^ and indeed one patient in the intervention arm in the trial reported by Raman et al.^10^ discontinued IVIG therapy due to the development of rashes and one patient in the intervention arm in the trial reported by Parikh et al.^14^ reported development of erythema. Of note, the two trials reported by Tabarsi et al.^12^ and Gharebaghi et al.,^9^ respectively, did not make available the safety outcomes.

Our study has several limitations. One of the limitations identified is the potential bias within the individual trials included in the meta‐analysis. To address this concern, sensitivity analyses were performed by excluding trials with a high risk of bias. Surprisingly, sensitivity analyses revealed that IVIG administration in hospitalized COVID‐19 patients did not significantly reduce mortality risk in this population. This finding highlights the potential influence of trial quality and bias on the overall results. It suggests that the observed reduction in mortality odds with IVIG administration may have been driven by trials with a higher risk of bias. In light of these limitations and the need for more definitive evidence, we recommend conducting larger randomized trials with rigorous study designs. In addition, it is important to acknowledge that the trials included in our meta‐analysis had relatively small sample sizes, which could limit the generalizability of the findings. However, it is worth noting that there were two recently published trials by Maor et al.^16^ and the INSIGHT study trial^17^ that contributed larger sample sizes to the overall analysis. These trials offer a more robust representation of the patient population and treatment effects, thereby enhancing the generalizability of the individual trial findings to some extent. Additionally, by aggregating the results of these trials into a meta‐analysis, the statistical power has been increased, although more large‐scale trials are still desirable.

## 5 CONCLUSION

Our meta‐analysis demonstrated that IVIG administration significantly reduced the risk of mortality among hospitalized COVID‐19 patients. Given that IVIG might mitigate cytokine storms in these patients, future trials could modify IVIG administration to target only those with biochemical evidence of inflammation. This approach could help to better support the mortality benefits of IVIG administration. Our study has important implications in the management of hospitalized COVID‐19 patients globally. We suggest larger RCTs to confirm our findings and demand better reporting of the safety outcomes in future trials to understand the risk‐benefit ratio of IVIG therapy in COVID‐19.

## AUTHOR CONTRIBUTIONS

**Dinesh Sangarran Ramachandram**: Conceptualization; data curation; formal analysis; validation; writing—original draft; writing—review and editing. **Chia Siang Kow**: Conceptualization; data curation; formal analysis; writing—original draft; writing—review and editing. **Syed Shahzad Hasan**: Formal analysis; validation; writing—original draft; writing—review and editing. **Kaeshaelya Thiruchelvam**: Formal analysis; writing—original draft; writing—review and editing.

## CONFLICT OF INTEREST STATEMENT

The authors declare no conflict of interest.

## TRANSPARENCY STATEMENT

The lead author, Kaeshaelya Thiruchelvam, affirms that this manuscript is an honest, accurate, and transparent account of the study being reported, that no important aspects of the study have been omitted; and that any discrepancies from the study as planned (and if relevant, registered) have been explained.

## Data Availability

The data sets generated during and/or analyzed during the current study are available from the corresponding author upon reasonable request.

## References

[1] Schwab I , Nimmerjahn F . Intravenous immunoglobulin therapy: how does IgG modulate the immune system? Nat Rev Immunol. 2013;13(3):176‐189. 23411799 10.1038/nri3401

[2] Ballow M . The IgG molecule as a biological immune response modifier: mechanisms of action of intravenous immune serum globulin in autoimmune and inflammatory disorders. J Allergy Clin Immunol. 2011;127(2):315‐323. 21185071 10.1016/j.jaci.2010.10.030

[3] Ballow M . Mechanisms of immune regulation by IVIG. Current Op Aller Clin Immunol. 2014;14(6):509‐515. 10.1097/ACI.0000000000000116 25337683

[4] Gelfand EW . Intravenous immune globulin in autoimmune and inflammatory diseases. N Engl J Med. 2012;367(21):2015‐2025. 23171098 10.1056/NEJMra1009433

[5] Buszko M , Park JH , Verthelyi D , Sen R , Young HA , Rosenberg AS . The dynamic changes in cytokine responses in COVID‐19: a snapshot of the current state of knowledge. Nature Immunol. 2020;21(10):1146‐1151. 32855555 10.1038/s41590-020-0779-1

[6] Xiang H , Cheng X , Li Y , Luo W , Zhang Q , Peng W . Efficacy of IVIG (intravenous immunoglobulin) for coronavirus disease 2019 (COVID‐19): a meta‐analysis. Int Immunopharmacol. 2021;96:107732. 34162133 10.1016/j.intimp.2021.107732 PMC8084608

[7] Moher D , Liberati A , Tetzlaff J , Altman DG , PRISMA Group . Preferred reporting items for systematic reviews and meta‐analyses: the PRISMA statement. PLoS Med. 2009;6(7):e1000097. 19621072 10.1371/journal.pmed.1000097 PMC2707599

[8] Sterne JAC , Savović J , Page MJ , et al. RoB 2: a revised tool for assessing risk of bias in randomised trials. BMJ. 2019;366:l4898. 31462531 10.1136/bmj.l4898

[9] Gharebaghi N , Nejadrahim R , Mousavi SJ , Sadat‐Ebrahimi SR , Hajizadeh R . The use of intravenous immunoglobulin gamma for the treatment of severe coronavirus disease 2019: a randomized placebo‐controlled double‐blind clinical trial. BMC Infect Dis. 2020;20(1):786. 33087047 10.1186/s12879-020-05507-4 PMC7576972

[10] Raman RS , Bhagwan Barge V , Anil Kumar D , et al. A phase II safety and efficacy study on prognosis of moderate pneumonia in coronavirus disease 2019 patients with regular intravenous immunoglobulin therapy. J Infect Dis. 2021;223(9):1538‐1543. 33585890 10.1093/infdis/jiab098 PMC7928808

[11] Sakoulas G , Geriak M , Kullar R , et al. Intravenous immunoglobulin plus methylprednisolone mitigate respiratory morbidity in coronavirus disease 2019. Critical Care Explorations. 2020;2(11):e0280. 33225306 10.1097/CCE.0000000000000280 PMC7671875

[12] Tabarsi P , Barati S , Jamaati H , et al. Evaluating the effects of intravenous immunoglobulin (IVIg) on the management of severe COVID‐19 cases: a randomized controlled trial. Int Immunopharmacol. 2021;90:107205. 33214093 10.1016/j.intimp.2020.107205 PMC7665876

[13] Ali S , Uddin SM , Shalim E , et al. Hyperimmune anti‐COVID‐19 IVIG (C‐IVIG) treatment in severe and critical COVID‐19 patients: a phase I/II randomized control trial. EClinicalMedicine. 2021;36:100926. 34109306 10.1016/j.eclinm.2021.100926 PMC8177439

[14] Parikh D , Chaturvedi A , Shah N , Patel P , Patel R , Ray S . Safety and efficacy of COVID‐19 hyperimmune globulin (HIG) solution in the treatment of active COVID‐19 infection‐findings from a prospective, randomized, controlled, multi‐centric trial. medRxiv. 2021.

[15] Mazeraud A , Jamme M , Mancusi RL , et al. Intravenous immunoglobulins in patients with COVID‐19‐associated moderate‐to‐severe acute respiratory distress syndrome (ICAR): multicentre, double‐blind, placebo‐controlled, phase 3 trial [published online ahead of print, 2021 Nov 11]. Lancet Respir Med. 2021;S2213‐2600(21):00440‐00449. 10.1016/S2213-2600(21)00440-9 PMC8585489 34774185

[16] Maor Y , Shinar E , Izak M , et al. A randomized controlled study assessing convalescent immunoglobulins vs convalescent plasma for hospitalized patients with coronavirus 2019. Clin Infect Dis. 2023;77(7):964‐971. 37220751 10.1093/cid/ciad305 PMC10552585

[17] Polizzotto MN , Nordwall J , Babiker AG , et al. ITAC (INSIGHT 013) Study Group . Hyperimmune immunoglobulin for hospitalised patients with COVID‐19 (ITAC): a double‐blind, placebo‐controlled, phase 3, randomised trial. Lancet. 2022;399(10324):530‐540. 35093205 10.1016/S0140-6736(22)00101-5 PMC8797010

[18] Shao Z , Feng Y , Zhong L , et al. Clinical efficacy of intravenous immunoglobulin therapy in critical ill patients with COVID‐19: a multicenter retrospective cohort study. Clin Transl Immunol. 2020;9(10):e1192. 10.1002/cti2.1192 PMC7557105 33082954

[19] Esen F , Özcan PE , Orhun G , et al. Effects of adjunct treatment with intravenous immunoglobulins on the course of severe COVID‐19: results from a retrospective cohort study. Curr Med Res Opin. 2021;37(4):543‐548. 33236646 10.1080/03007995.2020.1856058

[20] Galeotti C , Kaveri SV , Bayry J . Intravenous immunoglobulin immunotherapy for coronavirus disease‐19 (COVID‐19). Clin Transl Immunol. 2020;9(10):e1198. 10.1002/cti2.1198 PMC7565103 33088506

[21] Stiehm ER . Adverse effects of human immunoglobulin therapy. Transfus Med Rev. 2013;27(3):171‐178. 23835249 10.1016/j.tmrv.2013.05.004

[22] Pierce LR , Jain N . Risks associated with the use of intravenous immunoglobulin. Transfus Med Rev. 2003;17(4):241‐251. 14571392 10.1016/s0887-7963(03)00038-5

[23] Ballow M . Safety of IGIV therapy and infusion‐related adverse events. Immunol Res. 2007;38(1‐3):122‐132. 17917017 10.1007/s12026-007-0003-5

[24] Lozeron P , Not A , Theaudin M , et al. Safety of intravenous immunoglobulin in the elderly treated for a dysimmune neuromuscular disease. Muscle Nerve. 2016;53(5):683‐689. 26467654 10.1002/mus.24942

[25] Waheed W , Ayer GA , Jadoo CL , et al. Safety of intravenous immune globulin in an outpatient setting for patients with neuromuscular disease. Muscle Nerve. 2019;60(5):528‐537. 31443119 10.1002/mus.26678

[26] Guo Y , Tian X , Wang X , Xiao Z . Adverse effects of immunoglobulin therapy. Front Immunol. 2018;9:1299. 29951056 10.3389/fimmu.2018.01299 PMC6008653

[27] Wittstock M , Benecke R , Zettl UK . Therapy with intravenous immunoglobulins: complications and side‐effects. Eur Neurol. 2003;50(3):172‐175. 14530624 10.1159/000073059

[28] Ovanesov MV , Menis MD , Scott DE , et al. Association of immune globulin intravenous and thromboembolic adverse events. Am J Hematol. 2017;92(4):E44‐E45. 28066925 10.1002/ajh.24644

[29] Hasan SS , Radford S , Kow CS , Zaidi STR . Venous thromboembolism in critically ill COVID‐19 patients receiving prophylactic or therapeutic anticoagulation: a systematic review and meta‐analysis. J Thromb Thrombolysis. 2020;50(4):814‐821. 32748122 10.1007/s11239-020-02235-z PMC7396456

[30] Gerstenblith MR , Antony AK , Junkins‐Hopkins JM , Abuav R . Pompholyx and eczematous reactions associated with intravenous immunoglobulin therapy. J Am Acad Dermatol. 2012;66(2):312‐316. 21601310 10.1016/j.jaad.2010.12.034

